# A Real-Time Robust Method to Detect BeiDou GEO/IGSO Orbital Maneuvers

**DOI:** 10.3390/s17122761

**Published:** 2017-11-29

**Authors:** Guanwen Huang, Zhiwei Qin, Qin Zhang, Le Wang, Xingyuan Yan, Lihong Fan, Xiaolei Wang

**Affiliations:** College of Geology Engineering and Geomantic, Chang’an University, 126 Yanta Road, Xi’an 710054, China; huang830928@163.com (G.H.); rexlele@163.com (L.W.); yanxydice@163.com (X.Y.); fanlihong321@163.com (L.F.); gnssrwxl@126.com (X.W.)

**Keywords:** GEO/IGSO, real-time, orbital maneuver, time discrimination, satellite identification

## Abstract

The frequent maneuvering of BeiDou Geostationary Orbit (GEO) and Inclined Geosynchronous Orbit (IGSO) satellites affects the availability of real-time orbit, and decreases the accuracy and performance of positioning, navigation and time (PNT) services. BeiDou satellite maneuver information cannot be obtained by common users. BeiDou broadcast ephemeris is the only indicator of the health status of satellites, which are broadcast on an hourly basis, easily leading to ineffective observations. Sometimes, identification errors of satellite abnormity also appear in the broadcast ephemeris. This study presents a real-time robust detection method for a satellite orbital maneuver with high frequency and high reliability. By using the broadcast ephemeris and pseudo-range observations, the time discrimination factor and the satellite identification factor were defined and used for the real-time detection of start time and the pseudo-random noise code (PRN) of satellites was used for orbital maneuvers. Data from a Multi-GNSS Experiment (MGEX) was collected and analyzed. The results show that the start time and the PRN of the satellite orbital maneuver could be detected accurately in real time. In addition, abnormal start times and satellite abnormities caused by non-maneuver factors also could be detected using the proposed method. The new method not only improves the utilization of observations for users with the data effective for about 92 min, but also promotes the reliability of real-time PNT services.

## 1. Introduction

Since 27 December 2012, the BeiDou-2 regional navigation system has provided regional positioning, navigation and timing (PNT) services across the Asia-Pacific region. By 2020, the BeiDou-3 with 35 satellites in orbit will provide worldwide users with PNT services [1,2,3,4,5,6]. Navigation satellites are affected by Earth’s non-spherical gravity and other perceptual factors, leading to long-term perturbations of orbital elements and the offset of the satellite location. In order to keep the navigation satellite in the nominal orbital position, orbital maneuver is needed. It uses the propulsion systems to change the orbit of a satellite. GEO and IGSO satellites need to be frequently maneuvered to maintain their geosynchronous characteristics. After orbit maneuvering, satellite positions will vary by tens of kilometers, causing serious impacts on it services due to having to perform the positioning and orbit determination [7,8,9]. To adjust the strategies for positioning and orbit determination in a correct and timely manner, the abnormal conditions and maneuvers must be identified as soon as possible after they occur [10,11,12,13,14,15,16]. In practice, BeiDou satellite maneuver information is not available publicly, and only the hourly ephemeris broadcasts marks the satellite’s health status. Due to the low time-sampling rate and the identification errors of satellite abnormity of the BeiDou broadcast ephemeris, plenty of effective, useful observations are lost. Therefore, it is necessary to find a real-time detection method for satellite orbital maneuvers with high time-resolution and reliability.

Recently, Sciré et al. used space-based optical observation data to analyze the spatial debris orbit determination algorithm without the orbital maneuver detection [17]. Cui et al. used the orbital residual to detect the start time of the simulated GNSS orbital maneuver, but a complex mechanics model was needed [18]. Yan et al. analyzed a method to detect the maneuvering satellite using the restored orbit from the broadcast ephemeris of the BeiDou Navigation Satellite System (BDS) [19]. Ye et al. realized non-real time orbital maneuver detection using the Root-Mean-Square between the GEO and IGSO [20]. Su et al. used the mechanical energy difference of unit mass between space target and spacecraft to detect the orbital maneuver in non-real time, although the effectiveness of their method was restricted by the number of stations [21]. Du et al. monitored the orbital maneuver of the BeiDou GEO satellite using the orbit measurement data of the China Area Positioning System (CAPS), however, CAPS measurements are not available to common users [22]. Although some research has been done on the orbital maneuvers of the BeiDou and other GNSS constellations, additional measurement data and prior complex mechanics models are both restricted for the common user. Moreover, real-time maneuver detection is hardly realized. The maneuver analysis for the IGSO satellite is not as good. In order to solve the above problems, this study presents a robust real-time detection method for the satellite orbital maneuver only by using the broadcast ephemeris and the pseudo-range observations of a single BDS receiver. The proposed method, which does not need any additional data information and the orbit force model, can easily detect the orbital maneuver and orbital abnormities simultaneously, in real time. The computational burden of the proposed method is so small that it can be realized in common receiver hardware. In addition, the proposed method benefits both static and dynamic positioning users.

## 2. Materials and Methods

About 1.5 h before orbit maneuvering (this time difference is discussed further in Section 3.4), the state of the satellite in the broadcast ephemeris would be marked as 1 for unhealthy. This means the real-time satellite orbital parameters provided by the broadcast ephemeris are not usable for about 1.5 h. The utilization rate of the available satellite data is reduced as data is discarded for this period, and the health status in the broadcast ephemeris is often misidentified or missing (this condition is discussed in Section 3.5), thereby seriously affecting the availability of the satellites. Because the real-time pseudo-range observations can reflect the real distance between the stations without the influence of an orbital maneuver, a method using real-time single point positioning (SPP) based on pseudo-range observations is presented to detect the maneuver. This is a flexible method that can be implemented in any user terminal.

As the satellite orbit changes gradually in the process of an orbital maneuver, the mean square error of unit weight (MSE) and pseudo-range residual of the real-time pseudo-range SPP will continuously increase in the corresponding time period. This unique feature of MSE and the pseudo-range residual is used to detect the orbital maneuver in this study. The time discriminant factor and the satellite identification factor are defined to detect the maneuver start time and PRN of the maneuvering satellite.

### 2.1. Time Discriminant Factor of Satellite Orbit Maneuver

The least squares method is selected to estimate the parameters in real-time pseudo-range SPP in this study. The indirect adjustment mode is used, and the weight of observation is given by the sine of satellite elevation angle. During the period of the satellite orbit maneuvering, the orbit parameters of the broadcast ephemeris are no longer correct and the corresponding pseudo-range observations would have a gross error. Because the least-squares method has no ability to resist the gross error, the MSE would become larger. When the MSE ${\hat{\sigma }}_{0}$ increases to a threshold $\sigma_{Max}$, the observation data is considered to be abnormal. The ${\hat{\sigma }}_{0}$ is calculated by,

(1) $$V=B\hat{x}-l$$

(2) $${\hat{\sigma }}_{0}=\sqrt{\frac{V^{T}PV}{n-m}}$$

where B is the coefficient matrix of the corrections for the estimated parameters, $\hat{x}$ is the correction for the estimated parameters, l is a constant term, V is the correction for observations, P is the weight matrix, n is the total number of observations, m is the number of the estimated parameters.

The time discrimination factor (hereby referred to as “the time factor”) $\sigma_{M}\;$ of the satellite orbital maneuver is defined by the equation,

(3) $$\sigma_{M}\left(k\right)={\hat{\sigma }}_{0}\left(k\right)-\sigma_{Max}$$

where $\sigma_{Max}$ is the empirical threshold of MSE, k is the number of epochs. When $\sigma_{M}\left(k\right)$ is greater than 0 and keeps a sustained growth trend in the 10 min time period chosen for this study, the corresponding time of $\sigma_{M}\left(k\right)$ is considered to be the start time of the orbital maneuver.

### 2.2. The Satellite Identification Factor of Satellite Orbital Maneuver

The start time of the maneuver can be detected by the time factor, but the PRN number of the maneuvering satellite cannot be detected by this factor. Thus, another factor, the satellite identification factor (hereby referred to as “the satellite factor”) is defined and used to detect the PRN of the maneuvering satellite, which is calculated by the residuals of the pseudo-range. The distance $S_{i}^{j}\left(k\right)$ between the station and satellite is calculated by,

(4) $$S_{i}^{j}\left(k\right)=\sqrt{{\left(X^{j}\left(k\right)-X_{i}\left(k\right)\right)}^{2}+{\left(Y^{j}\left(k\right)-Y_{i}\left(k\right)\right)}^{2}+{\left(Z^{j}\left(k\right)-Z_{i}\left(k\right)\right)}^{2}}$$

where $X^{j}\left(k\right),\;Y^{j}\left(k\right),\;Z^{j}\left(k\right)$ are the spatial coordinates of the satellite *j* at epoch k; $X_{i},\;Y_{i},\;Z_{i}$ are the spatial coordinates of the station *i*; $S_{i}^{j}\left(k\right)$ is the distance calculated between the station and the satellite. After the correction of the satellite clock offset, the receiver clock offset, the ionospheric delay and the tropospheric delay, the corrected pseudo-range ${\hat{D}}_{i}^{j}\left(k\right)$ of the raw observations ${\tilde{D}}_{i}^{j}\left(k\right)\;$is:

(5) $${\hat{D}}_{i}^{j}\left(k\right)={\tilde{D}}_{i}^{j}\left(k\right)+c\delta t^{j}-c\delta t_{i}+\delta I_{i}^{j}\left(t\right)+\delta T_{i}^{j}\left(t\right)$$

where ${\tilde{D}}_{i}^{j}\left(k\right)$ is the pseudo-range observation of satellite j at epoch k; c is the speed of light; $\delta t^{j}$ is the satellite clock offset; $\delta t_{i}$ is the receiver clock offset; $\delta I_{i}^{j}$ is the correction of the ionospheric delay; and $\delta T_{i}^{j}$ is the correction of the tropospheric delay. Once the orbit maneuver occurs, ${\hat{D}}_{i}^{j}\left(k\right)$ is close to the true distance between the station and the satellite. However, $S_{i}^{j}\left(t\right)$ would have a gross error as a result of the wrong orbit parameters in the broadcast ephemeris. Then, the absolute residual value of pseudo-range $L_{i}^{j}\left(k\right)$ could be used for maneuver detection, which is calculated by:

(6) $$L_{i}^{j}\left(k\right)=\left|{\hat{D}}_{i}^{j}\left(k\right)-S_{i}^{j}\left(k\right)\right|$$

where, | | is the function of the absolute value. Like the time factor, the empirical threshold $L_{Max}^{j}$ of satellite *j* is given in advance. The satellite factor $L_{M}^{j}\left(k\right)$ of the satellite orbital maneuver is defined by:

(7) $$L_{M}^{j}\left(k\right)=L_{i}^{j}\left(k\right)-L_{Max}^{j}$$

$L_{Max}^{j}$ is the empirical threshold of the pseudo-range observations belonging to satellite *j*. With $L_{M}^{j}\left(k\right)$ greater than 0 and keeping a sustained growth trend in the period of 10 min chosen for this study, the corresponding satellite *j* is considered to be the maneuvering satellite.

### 2.3. Robust Equivalent Weight Matrix of the Observations

It needs to be emphasized that, when a satellite maneuvers, the orbital deviation would lead to two consequences. First is the increase in the MSE ${\hat{\sigma }}_{0}\left(k\right)$ discussed in Section 2.1, which can be used to detect the start time of the orbital maneuver. The other is the increase in the absolute residual value of pseudo-range, $L_{i}^{j}\left(k\right)$ (in Section 2.2), which is used to detect the PRN of the maneuvering satellite. However, the least squares method has no ability to resist the error. The $L_{i}^{j}\left(k\right)$ of all satellites would increase influenced by the deviation of the maneuver orbit which leads to difficulty in confirming the PRN of the maneuvering satellite. Thus, this study presents a method of robust equivalent weight matrix for observation [23]. The weight matrix for observation is adaptively adjusted according to the residual value in real-time. This method can avoid the disturbance of the orbit deviation to the $L_{i}^{j}\left(k\right)$ of non-maneuvering satellites. Considering the rapid changes of $L_{i}^{j}\left(k\right)$ after the orbital maneuvering, the two-stage function of the robust equivalent weight matrix is structured by:

(8) $${\bar{p}}^{j}\left(k\right)=\{\begin{array}{cc} p^{j}\left(k\right) & \left|\frac{L_{i}^{j}\left(k\right)}{L_{Max}^{j}}\right|\leq r\\ 0 & \left|\frac{L_{i}^{j}\left(k\right)}{L_{Max}^{j}}\right|>r \end{array}$$

where $p^{j}\left(k\right)$ is the diagonal element of the observation weight matrix P for *j* satellite at *k* epoch. ${\bar{p}}^{j}\left(k\right)$ is the diagonal element of the observation weight adjusted for robustness. r is the critical index assigned as 3.0 based on experience and the empirical threshold in this area, which is sensitive to the quality of station observations.

If one satellite *j*, is suspected as the maneuvering orbit at epoch *k*, it would be marked as 0 (1 for normal, 0 for doubtful maneuvering). Then, the pseudo-range SPP at the next epoch *k* + 1 would be continued with ${\bar{p}}^{j}\left(k+1\right)$ valued 0. Thus, the $L_{i}^{j}\left(k\right)$ of other non-maneuvering satellites marked as 1 would not be influenced. 

There are three steps to detect the orbital maneuvering using pseudo-range SPP at every epoch. Firstly, according to the maneuvering mark of the previous epoch *k* − 1, the $L_{i}^{j}\left(k\right)$ would be calculated after the pseudo-range SPP without a robust method. Secondly, the ${\bar{p}}^{j}\left(k\right)$ would be adjusted by Equation (8) for $L_{i}^{j}\left(k\right)$. Thirdly, the pseudo-range SPP would be calculated again with ${\bar{p}}^{j}\left(k\right)$. Then, the ${\hat{\sigma }}_{0}$ and the new $L_{i}^{j}\left(k\right)$ could be obtained to update the maneuvering mark and calculate $L_{M}^{j}\left(k\right)$ following Equation (7). The next epoch *k* + 1 would be continued following these 3 steps.

### 2.4. The Selection of Empirical Threshold

The selection of the empirical threshold for the time factor and the satellite factor is key in this method. The empirical threshold is chosen by statistical theory and experience. Specifically, the threshold of time factors of satellite orbit maneuvering is forecasted by the MSE of the observation equation from the previous data. The threshold of the satellite factor is forecasted by the residual of the pseudo-range from the previous data. The threshold of time factors at one station is the same for all GEO/IGSO satellites, as the internal precision is not easily influenced by weather or other factors. The threshold of the satellite factor at one station is different for GEO/IGSO satellites, as the pseudo-range observation is easily influenced by weather or other factors. Actually, the MSE of observation equations follows a Chi-square Distribution, and the residual of the pseudo-range follows a Normal Distribution. The MSE values in the normal condition are all in the confidence interval with a 95.44% (2σ) confidence coefficient. The residual of pseudo-range values in the normal condition of GEO and IGSO are all in the confidence interval with a 99.74% (3σ) confidence coefficient. That is, if the factor is outside of the corresponding confidence interval, it is considered to be abnormal. Thus, the $\sigma_{Max}$ is given by the upper confidence interval of MSE with a 95.44% confidence coefficient. The $L_{Max}$ for GEO and IGSO is given by the upper limit of the confidence interval of residuals of pseudo-range with a 99.74% confidence coefficient.

**Figure 1:**
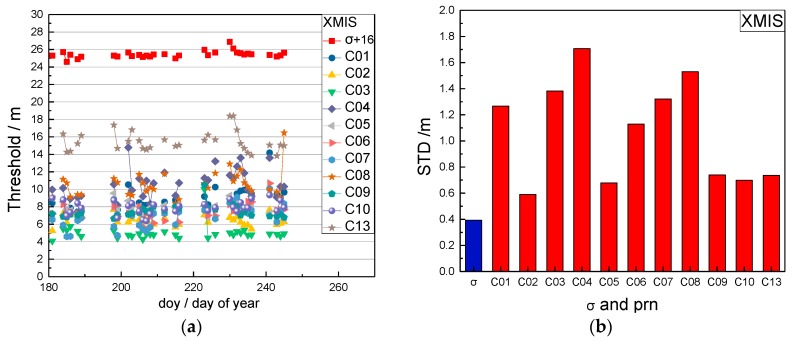
The threshold results of the station XMIS between day of year (doy) 181 and 245 in 2016. (**a**) The threshold series of the time factor $\sigma_{M}$ and the satellite factor $L_{M}$; (**b**) the standard deviation of the thresholds.

In order to analyze the robustness of the selected method for the above thresholds, the long-time BDS data of station XMIS (the station description is given in Section 3.1) was analyzed. The threshold results (one value per day) between doy (day of year) 181 and 245 in 2016 were chosen and are shown in Figure 1.

In Figure 1a, the red squares are the thresholds of the time factors which are added 16 m for plotting comparison. The other point-lines are the thresholds of the satellite factors for each satellite. Figure 1b gives the standard deviations of the thresholds. The blue bar is the standard deviation of the time factor thresholds and the red bars are the standard deviation of the satellite factor thresholds. On some days, data in Figure 1 was lost due to unavailable observations during this period.

Figure 1 shows that the calculated thresholds of the time factors change slightly and have a small standard deviation (0.4 m). The thresholds of the satellite factors for each satellite have differences and have greater standard deviations (0.6 m to 1.7 m), which are caused by the different accuracies of the broadcast ephemeris and the influence of weather or other factors. Considering that the thresholds of the time factors are steady during a longer time, and the thresholds of the satellite factors keep steady only for a short period, the threshold of the time factor is predicted and updated every week and the threshold of satellite factor is updated every 3 days.

The following steps were used for orbital maneuver detecting:

(1) The thresholds of the time factor and the satellite factor should be achieved by the above method in Section 2.1, Section 2.2 and Section 2.3
(2) The real-time ${\hat{\sigma }}_{0}$ is calculated. When the $\sigma_{M}$ is greater than 0 and shows a sustained growth trend in *k* epochs, that is, 20 epochs in this study, the first epoch with $\sigma_{M}$ greater than 0 is considered to be the start time of the orbit maneuver.
(3) Once the orbit maneuver is confirmed, the $L_{M}^{j}\left(k\right)$ is calculated. If $L_{M}^{j}\left(k\right)$ of one satellite is greater than 0 and keeps a sustained growth trend in *k* epochs, that is, 20 epochs in this study, this satellite is considered to be the maneuvering satellite.

## 3. Example

### 3.1. Data Description

**Figure 2:**
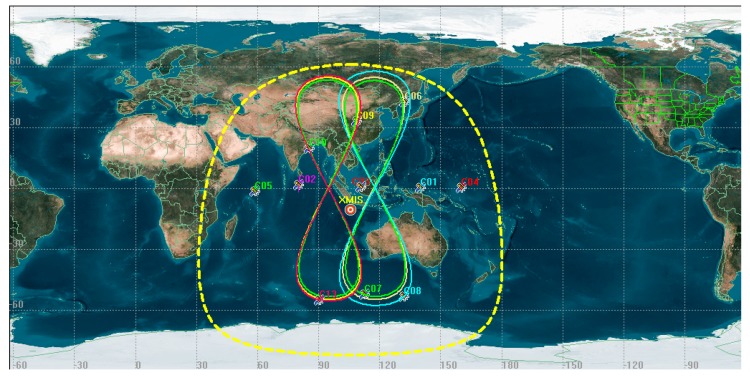
The data location and the distributions of the trajectories on the ground.

The data from the GNSS station XMIS from the Multi-GNSS Experiment (MGEX) was chosen to to be analyzed [24]. The station XMIS is located on Christmas Island, a territory of Australia (10°26′ S, 105°41′ E, see Figure 2), which is connected to a TRM59800.00 receiver that records data with a 30 s sampling period. The distributions of trajectories on station XMIS on doy 214 (1 August 2016) are shown in Figure 2.

The red circle indicates the location of the XMIS stations. The trajectories shown as an “figure 8” are for IGSO and the trajectories shown as points are for GEO. The satellites with of trajectories distributed within the yellow circle could be observed by XMIS.

The thresholds of the mean square error and the residuals of the pseudo-range for different satellites of the XMIS site on doy 200 (18 July 2016) and doy 214 (1 August 2016) are shown in Table 1.

Table 1 shows that the values of $\sigma_{Max}$ are similar and stable on different days, however the values of $L_{Max}$ for GEO/IGSO satellites are different and unstable. 

### 3.2. Orbital Maneuver Detection for GEO

**Figure 3:**
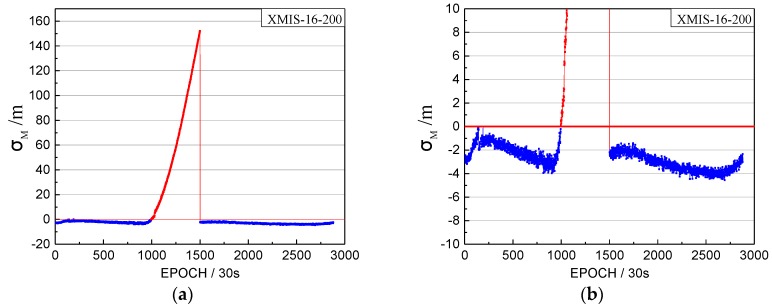
The time factor of the orbital maneuver for XMIS station on 18 July 2016. (**a**): The normal vertical axis scale shows overall information; (**b**): The amplified vertical axis scale shows more detailed information.

**Figure 4:**
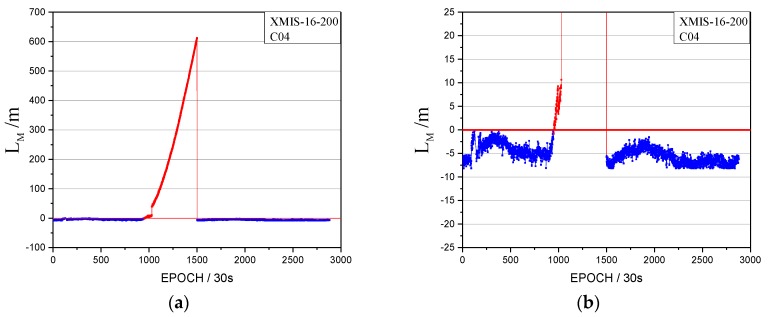
The satellite factor of the orbital maneuver for C04 on 18 July 2016. (**a**): The normal vertical axis scale shows overall information; (**b**): The amplified vertical axis scale shows more detailed information.

The orbital maneuver is detected for GEO on 18 July 2016, using the method in Section 2. The performance of the orbital maneuver detection for XMIS is given first, with the time factor series shown in Figure 3 and the satellite factor series shown in Figure 4. 

In Figure 3, the time factor series shows the sustained growth trend over about 4 h with values greater than 0 starting at epoch 995 (08:17). The start time of the orbital maneuver detected by the time factor on 18 July 2016 is 08:17. In Figure 9, the satellite factor series of satellite C04 shows the sustained growth trend over about 4 h with values greater than 0. Therefore, the PRN of the maneuvering satellite determined by the satellite factor is C04. 

In order to justify the correction of the detection results for XMIS, the health indicators, 1 for an unhealthy status and 0 for a healthy status, of BeiDou satellites in the broadcast ephemeris and information of precise orbit products provided by the iGMAS (International GNSS Monitoring and Assessment System) are used as a reference.

**Figure 5:**
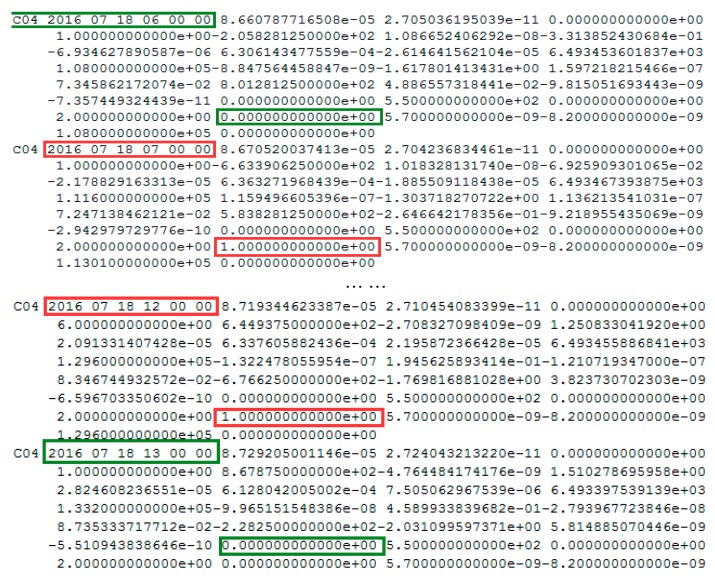
The health status of C04 in the broadcast ephemeris. The red box highlights the unhealthy status marked as 1. The blue box highlights the healthy status marked as 0. Note that the data is not displayed from between 8:00 and 11:00 to limit the size of the figure and the health status are all marked as 1 during this period.

**Figure 6:**
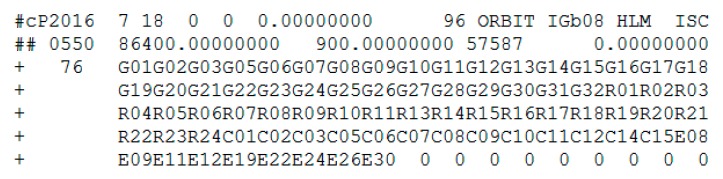
The header of the precise orbits information published by iGMAS on 18 July 2016.

The health status of C04 and the precise orbits products on 18 July 2016 are shown in Figure 5 and Figure 6, respectively.

In Figure 5, the health status of satellite C04 in the broadcast ephemeris is marked as unhealthy from 7:00:00 to 12:00:00. The PRN is correctly detected as C04 by the proposed method. The start time of the orbital maneuver detected for C04 is 08:17. As it is known to all, once the orbit maneuvers begin, the precision orbit cannot be determined because the orbital maneuver leads to kinetic empirical parameters failing. The header of the precise orbits results removed the C04 satellite as shown in Figure 6, which is secondary proof of the orbital maneuver for C04.

**Figure 7:**
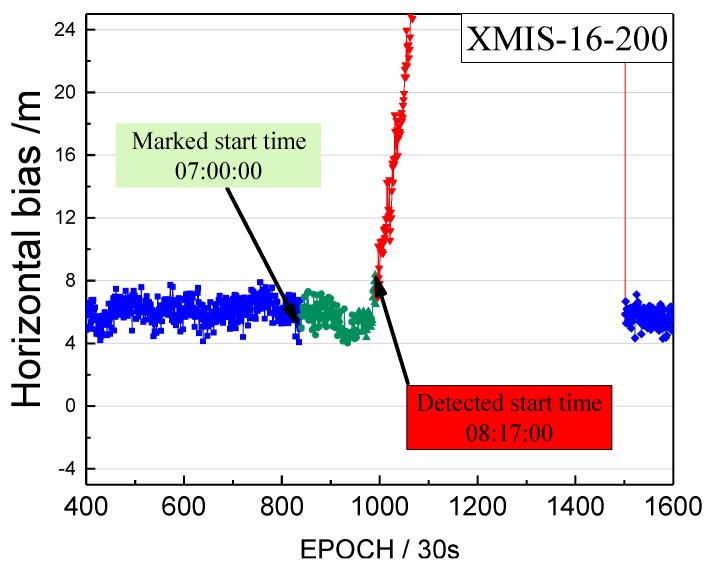
The bias between pseudo-range SPP coordinates and the SINEX coordinates (in horizontal) on doy 200, 2016.

Although the detected start moment of the orbital maneuver is marked as unhealthy, it also should be verified that the moment is close to the real start time of the orbital maneuver. Therefore, we calculated the bias between pseudo-range SPP coordinates and the SINEX coordinates from the IGS (International GNSS Service), to verify the correction of the detected start time. These biases of coordinates in a horizontal configuration are unified into one value of the point coordinates bias. This bias of XMIS on doy 200, 2016 is shown in Figure 7.

The detected start time of the orbital maneuver and the start time of unhealthy marks are indicated by the arrows. The blue line is the series before the unhealthy start time. The green line is the series between the detected start time and the unhealthy start time. The red line is the series after the detected start time.

The biases of the red section in Figure 7 shows an increasing trend greater than the biases of blue section, and the biases of the green section are almost equal to the biases of blue section. The observations between the detected start time and the unhealthy start time are usable and not influenced by the orbital maneuver. Section 2 mentions that the state of the satellite in the broadcast ephemeris would be marked as 1 about 1.5 h before the maneuvering, which leads to the loss of many usable observations. Figure 7 verifies that the loss of the usable data can be used to enhance the utilization rate. In addition, Figure 7 shows that the observations after the detected start time are influenced quickly by the orbital maneuver, rendering the pseudo-range SPP results unreliable. In addition, the moment (8:17:00) is definitely close to the real start time of the orbital maneuver.

### 3.3. Orbital Maneuver Detection for IGSO

**Figure 8:**
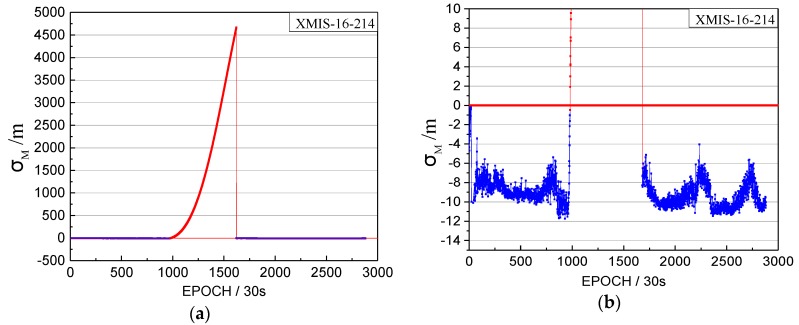
The time factors of the orbital maneuver for the XMIS station on 1 August 2016. (**a**) The normal vertical axis scale shows overall information; (**b**) The amplified vertical axis scale shows more detailed information.

**Figure 9:**
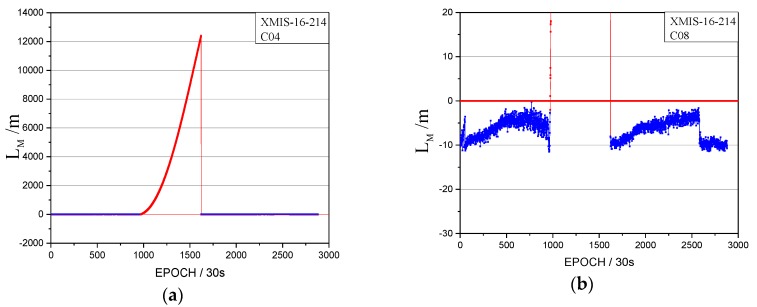
The satellite factors of the orbital maneuver for C08 on 1 August 2016. (**a**): The normal vertical axis scale shows overall information; (**b**): The amplified vertical axis scale shows more detailed information.

The orbital maneuver is detected for IGSO on 1 August 2016 using the proposed method in Section 2. The performance of the orbital maneuver detection is shown by the time factor series shown in Figure 8 and the satellite factor series shown in Figure 9.

When the time factor is greater than 0 and has a sustained growth trend for a period, this is considered to be the start time of the orbital maneuver. In Figure 8, the start time of the orbital maneuver detected by the time factor series on 1 August 2016 is epoch 972 (08:05:30). In Figure 9, the satellite factor series of satellite C08 show the sustained growth trend over about 4 h with values greater than 0. Therefore, the PRN of the maneuvering satellite determined by the satellite factor is C08.

**Figure 10:**
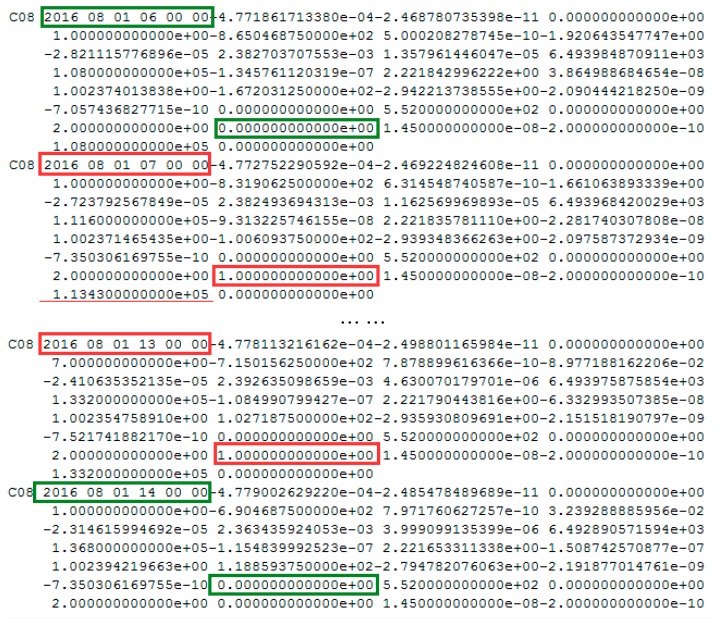
The health marks of C08 in the broadcast ephemeris. The red box highlights are the unhealthy status marked 1. The blue box highlights are the healthy status marked 0. Note the data from between 08:00 and 12:00 is not displayed to limit the size of the figure, and the healthy status are all marked 1 during this period.

**Figure 11:**
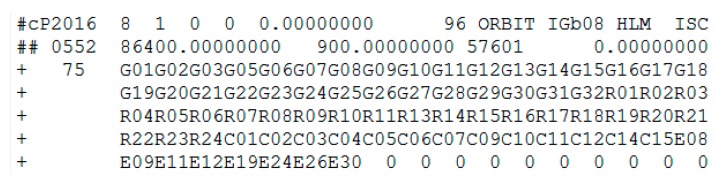
The header of precise orbits results of iGMAS on 1 August 2016.

The health marks of the BeiDou satellite for C08 and precise orbits results on 1 August 2016 are shown in Figure 10 and Figure 11, respectively.

In Figure 10, the health status of satellite C08 in the broadcast ephemeris is marked as unhealthy from 07:00 to 13:00. The PRN is correctly detected as C08 by the proposed method. The start time of the orbital maneuver detected for C08 is 08:05:30 (between 07:00 and 13:00). As is known, once the orbit maneuvers begin, the precision orbit cannot be determined because of the orbital maneuver leads to kinetic empirical parameters failing. The header of the precise orbits information removed the C08 satellite, as shown in Figure 11, which is secondary proof of the orbital maneuver for C08.

### 3.4 Orbital Maneuver Detection for GEO/IGSO in 2016

In order to verify the applicability of the proposed method for long-term orbital maneuver detection, the maneuver detection results of 2016 were calculated. The detected results are compared with the health marks of BeiDou satellites in the broadcast ephemeris. The detected satellites are all marked unhealthy in the period, including the corresponding detected start time, as shown in Table 2.

The detected satellites, the detected start times, the marked satellites and the marked start times are given. The time differences between the detected start times and the marked times are given.

Table 2 shows that the BeiDou GEO satellites had 58 orbital maneuvers in 2016 (15 times for C03, 14 times for C02, 12 times for C01, 10 times for C05, 7 times for C04) and IGSO satellites had 12 orbital maneuvers (2 times for C06, 2 times for C07, 2 times for C08, 3 times for C09, 2 times for C10, 1 times for C13). As the GEO satellite is limited to a certain area, it needs more maneuvers to resist the Earth’s non-spherical gravity and other perceptual factors. The maneuver frequency of IGSO is lower than that of GEO satellites. The average time difference between the detected start times and the marked start times is 92.2 min. The observations during these time differences are still usable. Therefore, the proposed method could flexibly detect orbit maneuvers in real-time and improve the utilization ratio of observations.

Specifically, in Table 2, the C03 health status of the broadcast ephemeris is marked for delaying about 45 min in doy 158. This means that the health statuses of BeiDou broadcast ephemeris are unreliable and not always advanced.

### 3.5. Another Application of the Detected Method

**Figure 12:**
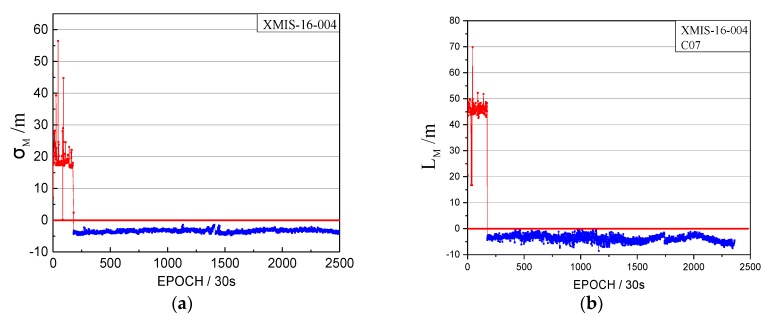
The abnormal time factor series and the satellite factor series. (**a**) The time factor series; (**b**) the satellite factor series.

When we analyzed the time factors and the satellite factors, an interesting abnormal situation appeared, as shown in Figure 12.

Note that the vertical coordinates are unified minus the threshold value, that is, when the factors are lower than 0, the observations tend to be normal.

**Figure 13:**
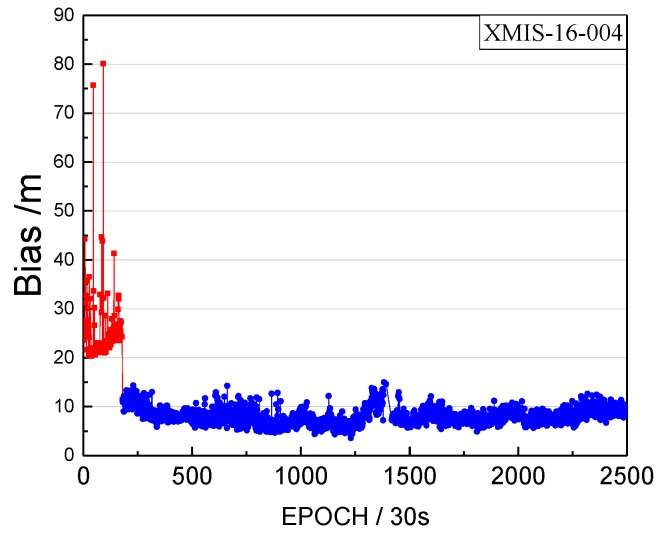
The bias of XMIS between pseudo-range SPP coordinates and the SINEX coordinates on doy 004, 2016.

**Figure 14:**
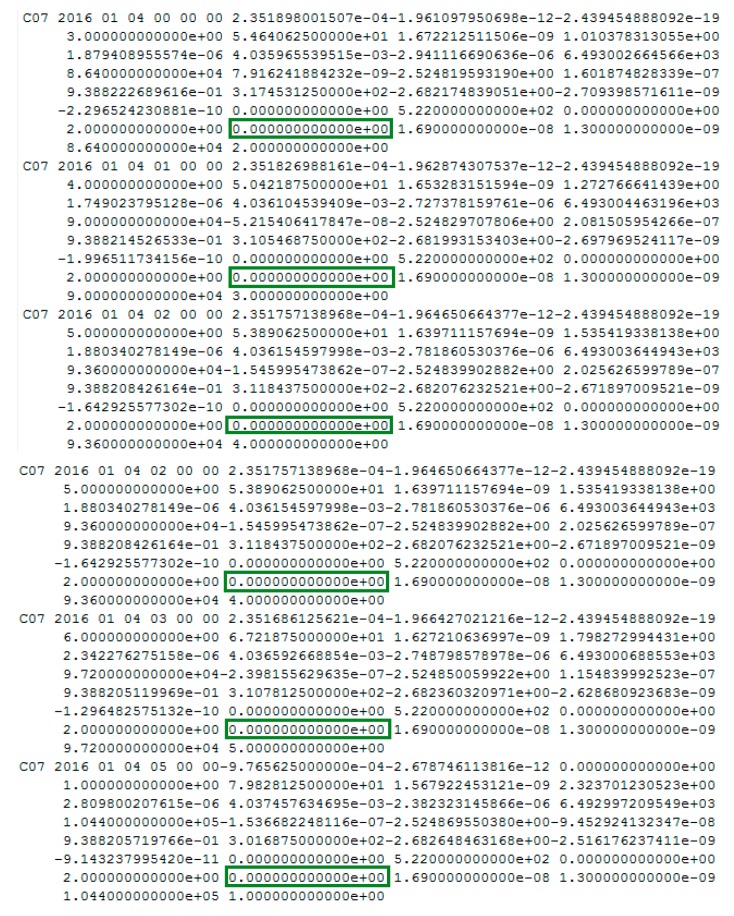
The health marks of C07 in the broadcast ephemeris. The green box highlights the health status marked 0 at the corresponding time of the abnormal status.

Figure 12 shows abnormal jumps in the time factors and the satellite factors series. Although the $\sigma_{Max}$ and $L_{Max}$ are both greater than 0, they cannot satisfy the factor series trend of the orbital maneuver. The pseudo-range SPP results are influenced by this abnormal situation, as shown in Figure 13. However, the abnormal status is not marked as unhealthy in the broadcast ephemeris, as shown in Figure 14.

The red section corresponds to the abnormal time period.

Figure 12, Figure 13 and Figure 14 indicate that the proposed method also could be used to detect abnormal orbit status changes. However, these abnormal orbit statuses are not depicted in the broadcast ephemeris. Specifically, if the $\sigma_{M}$ is greater than 0 and shows abnormal jumps in *k* epochs, which can be detected by one and two derivatives, then the first epoch with $\sigma_{M}$ greater than 0 is considered to be the start time of the abnormal status. Once the abnormal status is detected, the $L_{0}$ is calculated. If the $L_{M}$ of one satellite is greater than 0 and shows abnormal jumps in *k* epochs, this satellite is considered to be the satellite with an abnormal status.

### 3.6. The Application of the Detected Method for other GNSS MEO Constellations

In order to verify the applicability of this method for maneuvering in other GNSS MEO constellations, the detection results of GPS satellite maneuvering by station CANT (located in Cantabria, Spain 43° 28′ N, 356° 12′ E) are as follows.

**Figure 15:**
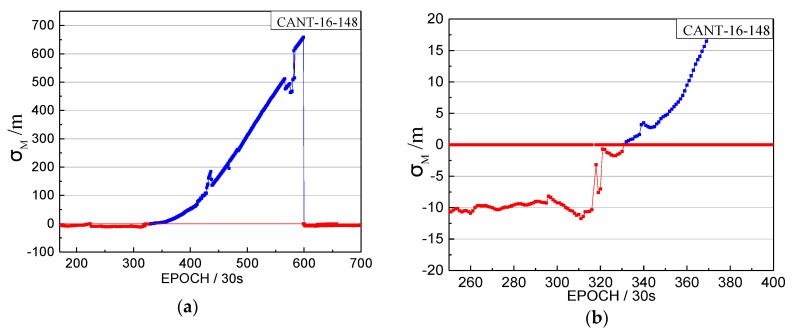
The time factors of the orbital maneuver for CANT station on 27 May 2016. (**a**) The normal vertical axis scale showing overall information; (**b**) The amplified vertical axis scale showing more detailed information.

**Figure 16:**
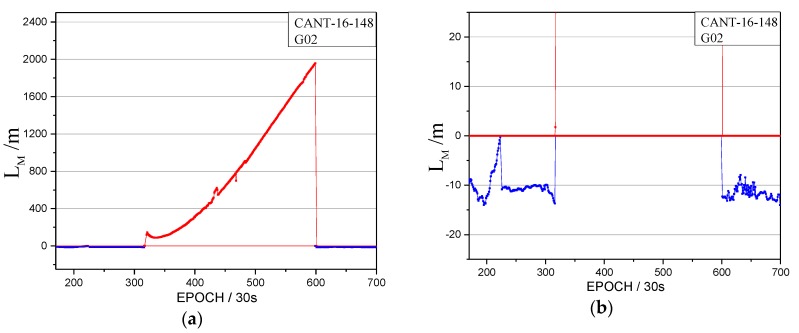
The satellite factors of the orbital maneuver for G02 on 27 May 2016. (**a**): The normal vertical axis scale shows overall information; (**b**): The amplified vertical axis scale shows more detailed information.

The orbital maneuver is detected for MEO on 27 May 2016 using the proposed method in Section 2. The performance of orbital maneuver detection is shown by the time factor series in Figure 15 and the satellite factor series in Figure 16.

When the time factor is greater than 0 and has a sustained growth trend in a period, this is considered to be the start time of the orbital maneuver. In Figure 15, the start time of the orbital maneuver detected by the time factor series on 27 May 2016 is epoch 231 (02:45). In Figure 16, the satellite factor series of satellite G02 show the sustained growth trend over about 2.5 h with values greater than 0. Therefore, the PRN of the maneuvering satellite determined by the satellite factor is G02.

The health marks of the GPS satellite for G02 and the precise orbits results on 27 May 2016 were collected and analyzed, which is similar to that of Section 3.2. By comparing these with public GPS broadcast ephemeris and precise orbits information, it was found that the health status of satellite G02 in the broadcast ephemeris is marked as unhealthy from 01:59:44 to 06:00. The PRN is correctly detected as G02 by the proposed method. The start time of the orbital maneuver detected for G02 is 02:45:00 (between 01:59:44 and 06:00:00), which improves the utilization of observations for GPS users with the effective data in about 45 min. The header of the precise orbits information removed the G02 satellite, which is secondary proof of the orbital maneuver for G02. Therefore, the proposed detection method also can be used to detect the orbital maneuvers of other GNSS MEO constellations. However, because of the characteristics of the global orbit, the continuous orbital maneuver detection of the MEO satellite needs more than one station.

## 4. Conclusions and Discussion

This study presented a real-time orbit maneuvering detection method which uses the pseudo-range observations and the broadcast ephemeris. The data from MGEX stations was analyzed, and the results showed that the start time of the satellite orbital maneuver and the PRN of the satellite orbital maneuver could both be detected accurately. The average time difference between the detected start time and the marked start time in the broadcast ephemeris is 92.2 min for GEO and IGSO satellites in 2016, which means that the proposed method can extend the usable observations more than 1.5 h. In addition, the proposed method also could be used to detect abnormal situations except orbit maneuvers, which are not marked unhealthy in the broadcast ephemeris. 

In the end, the proposed method is very flexible and can be realized in any BDS receiver. It could clearly improve PNT services when orbit maneuvers occur and it is abnormal. The detection method also can be used to detect the orbital maneuvers of other GNSS MEO constellations.

## Figures and Tables

**Table 1:** The thresholds of the orbital maneuver detection (unit: meter).

Date	The Thresholds of XMIS Station on 18 July 2016.					The Thresholds of XMIS Station on 1 August 2016.				
$\sigma_{Max}$	9.20					9.47				
$L_{Max}$	C01 *	C02 *	C03 *	C04 *	C05 *	C01 *	C02 *	C03 *	C04 *	C05 *
	7.65	6.25	4.43	8.19	7.59	8.56	6.70	4.26	11.91	8.17
	C06	C07	C08	C09	C10	C06	C07	C08	C09	C10
	8.24	4.73	10.79	7.06	6.72	7.96	7.22	11.78	7.58	7.75
	C13	-	-	-	-	C13	-	-	-	-
	14.69	-	-	-	-	15.68	-	-	-	-

* The marked PRN with “*” is the satellite for GEO, and the unmarked PRN is the satellite for IGSO.

**Table 2:** Results of orbital maneuver detection in 2016 for BeiDou GEO/IGSO satellites. (Unit: hour:minute:second).

PRN	Doy	Marked Time	Detect Time	Difference	PRN	Doy	Marked Time	Detect Time	Difference
C01	9	4:00:00	5:49:30	1:49:30	C03	12	7:00:00	8:34:30	1:34:30
	45	4:00:00	5:53:30	1:53:30		40	7:00:00	8:38:00	1:38:00
	77	4:00:00	5:38:30	1:38:30		67	8:00:00	9:16:00	1:16:00
	105	9:00:00	10:55:00	1:55:00		92	7:00:00	9:42:00	2:42:00
	130	3:00:00	4:41:00	1:41:00		116	7:00:00	8:55:30	1:55:30
	165	4:00:00	5:43:30	1:43:30		144	10:00:00	11:21:30	1:21:30
	196	4:00:00	6:00:00	2:00:00		158	5:00:00	4:20:00	-0:40:00
	225	11:00:00	11:22:00	0:22:00		159	6:00:00	7:42:30	1:42:30
	252	10:00:00	11:22:00	1:22:00		183	8:00:00	9:27:00	1:27:00
	279	9:00:00	10:26:00	1:26:00		211	8:00:00	9:45:30	1:45:30
	309	7:00:00	8:23:30	1:23:30		238	8:00:00	9:49:30	1:49:30
	341	4:00:00	5:51:30	1:51:30		265	8:00:00	9:39:00	1:39:00
C02	**33**	11:00:00	12:47:00	1:47:00		291	8:00:00	9:38:00	1:38:00
	**52**	17:00:00	19:26:00	2:26:00		319	8:00:00	9:22:30	1:22:30
	**62**	0:00:00	1:17:30	1:17:30		348	7:00:00	8:25:30	1:25:30
	**95**	13:00:00	14:55:30	1:55:30	C04	88	7:00:00	8:53:30	1:53:30
	**125**	6:00:00	7:50:00	1:50:00		119	2:00:00	3:50:00	1:50:00
	**141**	9:00:00	11:09:30	2:09:30		200	7:00:00	8:17:00	1:17:00
	**168**	0:00:00	2:30:30	2:30:30		273	6:00:00	8:19:30	2:19:30
	**179**	2:00:00	8:30:00	6:30:00		312	14:00:00	15:21:00	1:21:00
	**180**	5:00:00	9:17:30	4:17:30		313	0:00:00	1:04:30	1:04:30
	**190**	0:00:00	1:04:30	1:04:30			7:00:00	8:22:30	1:22:30
	**217**	8:00:00	9:51:00	1:51:00	C06	134	20:00:00	21:00:00	1:00:00
	**262**	8:00:00	10:22:00	2:22:00		302	6:00:00	7:17:00	1:17:00
	**306**	8:00:00	10:08:30	2:08:30	C07	138	0:00:00	0:40:00	0:40:00
	**351**	7:00:00	9:47:00	2:47:00		316	9:00:00	10:10:00	1:10:00
C05	**3**	0:00:00	1:10:30	1:10:30	C08	52	3:00:00	4:37:30	1:37:30
	**36**	0:00:00	1:11:00	1:11:00		214	7:00:00	8:05:00	1:05:00
	**71**	0:00:00	1:09:30	1:09:30	C09	28	11:00:00	11:56:00	0:56:00
	**109**	0:00:00	1:24:00	1:24:00		84	23:00:00	0:45:30	1:45:30
	**148**	0:00:00	1:04:00	1:04:00		270	8:00:00	9:38:30	1:38:30
	**187**	0:00:00	1:09:00	1:09:00	C10	172	0:00:00	1:10:00	1:10:00
	**222**	0:00:00	0:53:30	0:53:30		344	10:00:00	10:05:00	0:05:00
	**256**	0:00:00	0:47:00	0:47:00	C13	229	0:00:00	1:20:00	1:20:00
	**294**	23:00:00	0:00:00	1:00:00	Average time differences between marked and detected				1:32:12
	**333**	0:00:00	0:55:30	0:55:30					
